# Mating with Stressed Males Increases the Fitness of Ant Queens

**DOI:** 10.1371/journal.pone.0002592

**Published:** 2008-07-02

**Authors:** Alexandra Schrempf, Jürgen Heinze

**Affiliations:** Biologie I, Universität Regensburg, Regensburg, Germany; University of Edinburgh, United Kingdom

## Abstract

**Background:**

According to sexual conflict theory, males can increase their own fitness by transferring substances during copulation that increase the short-term fecundity of their mating partners at the cost of the future life expectancy and re-mating capability of the latter. In contrast, sexual cooperation is expected in social insects. Mating indeed positively affects life span and fecundity of young queens of the male-polymorphic ant *Cardiocondyla obscurior*, even though males neither provide nuptial gifts nor any other care but leave their mates immediately after copulation and die shortly thereafter.

**Principal Findings:**

Here, we show that mating with winged disperser males has a significantly stronger impact on life span and reproductive success of young queens of *C. obscurior* than mating with wingless fighter males.

**Conclusions:**

Winged males are reared mostly under stressful environmental conditions, which force young queens to disperse and found their own societies independently. In contrast, queens that mate with wingless males under favourable conditions usually start reproducing in the safety of the established maternal nest. Our study suggests that males of *C. obscurior* have evolved mechanisms to posthumously assist young queens during colony founding under adverse ecological conditions.

## Introduction

During mating, males of many solitary insects transfer substances that increase the short-term fecundity of their mates at the cost of their future life expectancy and re-mating capability [1], [2]. In contrast, males of social insects are expected to benefit from increasing the life span of their mates, because these need to produce large numbers of sterile workers before they begin rearing sexuals and also do not re-mate later in life [3], [4]. Indeed, a recent study showed that in the ant *Cardiocondyla obscurior* mating itself is beneficial for females even though males neither provide nuptial gifts nor behaviourally assist young queens during colony founding in any other way. Mated queens lived longer, even when they had mated with sterilized males and had a similarly low egg laying rate as virgin queens [5].

**Figure 1:**
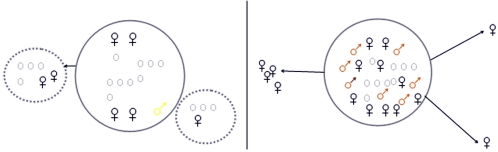
Different modes of colony foundation of *C. obscurior* queens. Under favourable conditions (left), wingless males (yellow male symbol) develop and mate with virgin queens (black female symbol), which start egg laying within the colony, assisted by workers (grey circles) and other queens (black female symbol). New colonies are founded by several individuals, which leave the mother colony and colonize new nest sites close by. Under stressful conditions (right), winged disperser males develop (brown male symbol) and mate with the virgin queens in and away from the maternal nest. Under such conditions, young queens leave the colony to colonize new nest sites in better environmental conditions and found either alone or together with other queens.

*C. obscurior* is a small myrmicine ant that lives in ephemeral nest sites, such as rolled-up leaves or cavities in wood [6]. Like many plants and other sessile organisms living in unpredictable environments, the ant species *C. obscurior* has evolved alternative reproductive tactics that allow the colony to flexibly react to habitat changes: under favourable environmental conditions, young queens mate with wingless fighter males inside the maternal nest, and, assisted by workers, quickly begin to produce new workers and sexuals to increase the size of the maternal colony. Large colonies eventually bud and spread to empty nest sites close by [6]. In contrast, colonies produce large numbers of virgin queens and-along with a few wingless males–numerous winged disperser males when conditions deteriorate, e.g. after sudden temperature drops, starvation, or an experimental reduction of worker numbers [7], [8]. Winged males mate inside the nest with virgin queens, but later leave the colony to mate with females from other nests. Under such stressful conditions, local budding is not profitable, and young queens instead benefit from dispersing and founding their nests away from the maternal colonies (Figure 1). Colony founding by dispersing queens requires much more time before sexuals are produced than budding by non-dispersing, local queens, as dispersing queens have to build up their own workforce before they can start with the production of new sexuals.

We thus expect that winged males, which are produced only under stressful conditions, should increase the colony founding success of their mates even more strongly than do wingless males, which are regularly produced under favourable conditions. Here, we show that mating with winged males has a stronger positive impact on colony founding success and longevity of queens than mating with wingless males.

## Results

Queens of *C. obscurior* are short-lived compared to other ant queens, which allowed us to investigate the complete life expectancy of queens mated with winged and wingless males. As single queens are usually not capable of founding a colony in the laboratory, we established groups of four queens. Such founding associations appear to be an alternative option for queens in the field [9].

**Figure 2:**
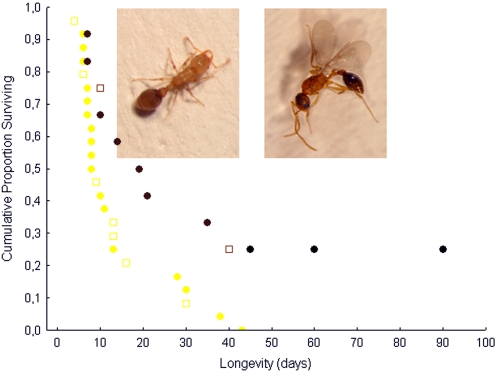
Lifespan of groups of *Cardiocondyla* queens. Groups of queens, which all mated with winged males (right, brown symbols), lived on average significantly longer than groups of queens, which all mated with a wingless male (left, yellow symbols), independent of whether queens were reared under stressed (filled dots) or unstressed conditions (open rectangles). Black dots indicate censored data, e.g., groups of queens still alive at the end of the experiment.

Groups of queens mated with winged males (n = 12 groups, 48 queens) survived significantly longer than groups of queens mated with wingless males (n = 24 groups, 96 queens), regardless of whether queens came from stressed or unstressed colonies (median, quartiles, range: winged: 20 days, 10, 42.5, 7–90 days, wingless: 8.5 days, 7, 14.5, 7–43 days; Cox's regression: male morph: B = −1.19, p = 0.01, queen rearing conditions: 0.31, p = 0.43; interaction term between male morph and queen rearing conditions: F = 0.46, p = 0.50; Figure 2) and also had a significantly higher probability of starting to lay eggs (eggs laid in 5 of 24 groups of queens mated with wingless males vs. 8 of 12 groups of queens mated with winged males, Yates corrected χ^2^ = 5.43, p<0.02). Although queen lifespan was very short, several queens managed to produce workers, which after the queens' death reared male and female sexuals from the queens' brood and thus guaranteed the continuation of the new colony. Because of their higher probability of egg laying, considerably more queens were able to raise workers when mated with the winged morph. Workers were produced in 2 of 24 groups of queens mated with wingless males (8.3%) and 4 of 12 groups of queens mated with winged males (33.3%, χ^2^ = 3.60, p<0.06).

**Figure 3:**
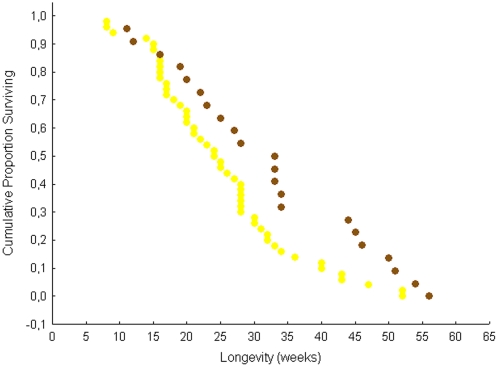
Lifespan of *Cardiocondyla* queens kept with 20 workers each. Unstressed queens that were assisted by workers lived on average significantly longer when mated with a winged male (brown) in comparison to queens which mated with a wingless male (yellow).

When freshly mated *C. obscurior* queens from unstressed colonies were assisted in colony founding by 20 workers each, queen life span was considerably longer. This second experimental set-up clearly documents the advantage of budding, but here again, queens mated with winged males (n = 22) lived significantly longer than queens, which mated with wingless males (n = 50; median, quartiles, range: winged 231 days, 154.0, 315.0, 56–364 days; wingless 164.5, 119.0, 217.0, 77–392 days; Cox's F-test: F(100, 44) = 1.802, p = 0.015; Figure 3). A comparison with previously published data on the life span of virgin *C. obscurior* queens kept together with 20 workers under exactly the same conditions [5] corroborates the conclusion that mating itself prolongs the life span (virgin queens, n = 34, median, quartiles, range: 136.5 days, 70, 175, 35–294 days), but mating with winged males has a significantly stronger impact than mating with wingless males. The morph of the queen's mating partner did not affect the onset of egg laying, egg laying rates, the beginning and rate of sexual production (t-tests, p>0.1) and sex ratio (Mann Whitney U-tests, p>0.5), but because queen life span and the number of sexuals produced were significantly correlated (Pearson's r = 0.43, p<0.001), queens that mated with a winged male reached considerably higher lifetime reproductive success (maximum 270 vs. 156 sexuals).

**Figure 4:**
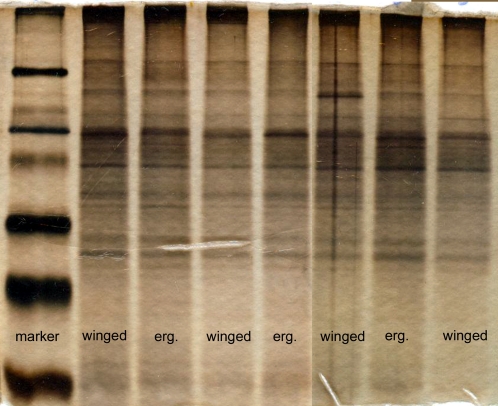
Electrophoresis pattern of the accessory gland extracts after silver staining. No differences in the protein pattern of pairs of glands of winged males (winged) and ergatoid males (erg.) could be detected.

To determine whether the difference between the effects of mating with winged vs. wingless males might depend on the quality or quantity of male accessory gland proteins, we examined the protein content and size of these glands. We could not detect any difference in the electrophoretic pattern of male seminal fluids between ergatoid and winged males (Figure 4). However, winged males are larger than wingless males, and their accessory glands are also significantly larger than those of wingless males (accessory gland size (area) mean±sd: wingless males, n = 9, 11277±1152 µm^2^; winged males, n = 9, 15229±1040 µm^2^; t-test: t = 7.64, p<0.001).

## Discussion

Winged males of *C. obscurior* appear to posthumously increase the founding success of queens, independent of their mode of colony founding. Mating with winged males prolonged the lifespan of queens, which were forced to disperse and start new colonies without assistance from workers long enough to allow the successful independent initiation of colonies away from the maternal nest.

Ant males neither provide nuptial gifts nor do they care for their mate or the brood [3], [4] but instead die after a number of copulations. The prolongation of the life span of *C. obscurior* queens by mating with winged males thus can only be explained either by queens detecting the presence or absence of wings in their mates and accordingly adjusting their life-history strategy, by the act of mating itself, or the quantity or quality of substances transferred by the male during copulation proximately affecting life expectancy. Winged and wingless males neither differed in behaviour during precopulatory courtship nor in behaviour and duration of the copulation itself [10]. Wingless males engage in precopulatory courtship for a few seconds longer than winged males [10], but this does presumably also cannot explain our result. Similarly, our study did not reveal any differences between the two male morphs in the electrophoretic pattern of seminal fluid proteins from their accessory glands. Though, our data reveal that the accessory glands of winged *C. obscurior* males are significantly larger than those of wingless males. Given that male seminal fluids have repeatedly been shown to affect the physiology of arthropod females [11]–[13] and that this impact varies with fluid quantity [14], winged *C. obscurior* males might provision queens with a higher amount of beneficial accessory gland products than wingless males and through this cooperative act increase the queen's probability of raising offspring under stressful conditions.

## Materials and Methods

A total of 140 young queens from stressed (colonies that produced winged males due to temperature drop or splitting) and unstressed laboratory colonies (colonies kept under standard conditions, which produced wingless males) were allowed to mate either with a non-nestmate winged male (from stressed colonies) or a non-nestmate wingless male (from stressed or unstressed colonies). As colony founding by single queens failed in the laboratory, we established groups of four queens by merging queens from different colonies but from the same rearing conditions (stressed and unstressed), each mated with a different male, but all with the same male morph (n = 12 mated with a winged male, n = 24 mated with a wingless male). These groups were monitored 5 days/week for the presence of eggs or larvae and the survival of queens.

In a second experiment, freshly emerged queens from unstressed colonies were allowed to either mate with a winged (n = 22) or a wingless male (n = 50) from another colony as described above. Afterwards, each queen was put together with 20 workers into a nest chamber, imitating colony founding by “budding”. Again, colonies were monitored for the presence of larvae and the survival of queens 5 days/week.

Proteins from male accessory glands were separated by electrophoresis on 12.5% polyacrylamid gels (SDS-Page; 10 cm×7.5 cm×7,5 µm ) after Lämmli [15]. We dissected the paired accessory glands out of males, transferred them into an Eppendorf cup with 10 µl dd H_2_O on ice and pierced them with forceps to free the secretions from the gland. The samples were diluted in 10 µl SDS–PAGE sample buffer and boiled for 3 minutes, spun at 14000×g for 2 minutes and loaded onto the polyacrylamid gels. After electrophoresis (1,5 h, 15mA, 180 V), proteins were visualized by silver staining [16]

The reproductive system of nine winged and wingless males each was dissected and their accessory glands photographed. Afterwards, the area of the accessory glands was measured with the computer program Image Analysis.
